# Genomic Characterization of Host Factors Related to SARS-CoV-2 Infection in People with Dementia and Control Populations: The GR@ACE/DEGESCO Study

**DOI:** 10.3390/jpm11121318

**Published:** 2021-12-07

**Authors:** Itziar de Rojas, Isabel Hernández, Laura Montrreal, Inés Quintela, Miguel Calero, Jose Luís Royo, Raquel Huerto Vilas, Antonio González-Pérez, Emilio Franco-Macías, Juan Macías, Manuel Menéndez-González, Ana Frank-García, Mónica Diez-Fairen, Carmen Lage, Sebastián García-Madrona, Nuria Aguilera, Pablo García-González, Raquel Puerta, Oscar Sotolongo-Grau, Silvia Alonso-Lana, Alberto Rábano, Alfonso Arias Pastor, Ana Belén Pastor, Anaïs Corma-Gómez, Angel Martín Montes, Carmen Martínez Rodríguez, Dolores Buiza-Rueda, Maria Teresa Periñán, Eloy Rodriguez-Rodriguez, Ignacio Alvarez, Irene Rosas Allende, Juan A. Pineda, María Bernal Sánchez-Arjona, Marta Fernández-Fuertes, Silvia Mendoza, Teodoro del Ser, GR@ACE/DEGESCO Consortium, Guillermo Garcia-Ribas, Pascual Sánchez-Juan, Pau Pastor, María J. Bullido, Victoria Álvarez, Luis M. Real, Pablo Mir, Gerard Piñol-Ripoll, Jose María García-Alberca, Miguel Medina, Adelina Orellana, Chris R. Butler, Marta Marquié, María Eugenia Sáez, Ángel Carracedo, Lluís Tárraga, Mercè Boada, Agustín Ruiz

**Affiliations:** 1 Research Center and Memory Clinic, Ace Alzheimer Center Barcelona—Universitat Internacional de Catalunya, 08017 Barcelona, Spain; iderojas@fundacioace.org (I.d.R.); ihernandez@fundacioace.org (I.H.); lmontrreal@fundacioace.org (L.M.); naguilera@fundacioace.org (N.A.); pgarcia@fundacioace.org (P.G.-G.); rpuerta@fundacioace.org (R.P.); osotolongo@fundacioace.org (O.S.-G.); salonso@fundacioace.org (S.A.-L.); aorellana@fundacioace.org (A.O.); christopher.butler@imperial.ac.uk (C.R.B.); mmarquie@fundacioace.org (M.M.); ltarraga@fundacioace.org (L.T.); mboada@fundacioace.org (M.B.); 2 CIBERNED, Network Center for Biomedical Research in Neurodegenerative Diseases, National Institute of Health Carlos III, 28220 Madrid, Spain; mcalero@isciii.es (M.C.); afrankg@gmail.com (A.F.-G.); clage@idival.org (C.L.); arabano@fundacioncien.es (A.R.); angel.mar.mon@hotmail.com (A.M.M.); buizarueda@gmail.com (D.B.-R.); teresaperinantocino@gmail.com (M.T.P.); eloymrod@gmail.com (E.R.-R.); pascualjesus.sanchez@scsalud.es (P.S.-J.); mjbullido@cbm.csic.es (M.J.B.); pmir@us.es (P.M.); jmgalberca@ianec.com (J.M.G.-A.); mmedina@ciberned.es (M.M.); 3 Grupo de Medicina Xenómica, Centro Nacional de Genotipado (CEGEN-PRB3-ISCIII), Universidade de Santiago de Compostela, 15705 Santiago de Compostela, Spain; ines.quintela@usc.es (I.Q.); angel.carracedo@usc.es (Á.C.); 4 UFIEC, Instituto de Salud Carlos III, 28220 Madrid, Spain; 5 CIEN Foundation/Queen Sofia Foundation Alzheimer Center, 28220 Madrid, Spain; abpastor@fundacioncien.es; 6 Depatamento de Especialidades Quirúrgicas, Bioquímica e Inmunología, Facultad de Medicina, Universidad de Málaga, 29016 Málaga, Spain; jlroyo@uma.es; 7 Unitat Trastorns Cognitius, Hospital Universitari Santa Maria de Lleida, 25198 Lleida, Spain; rhuerto@gss.cat (R.H.V.); aarias@gss.cat (A.A.P.); gerard_437302@hotmail.com (G.P.-R.); 8 Institut de Recerca Biomedica de Lleida (IRBLLeida), 25198 Lleida, Spain; 9 CAEBI, Centro Andaluz de Estudios Bioinformáticos, 41013 Sevilla, Spain; agonzalez@caebi.es (A.G.-P.); mesaez@caebi.es (M.E.S.); 10 Unidad de Demencias, Servicio de Neurología y Neurofisiología, Instituto de Biomedicina de Sevilla (IBiS), Hospital Universitario Virgen del Rocío/CSIC/Universidad de Sevilla, 41013 Sevilla, Spain; efranco17@gmail.com (E.F.-M.); bernalsanchezarjona@gmail.com (M.B.S.-A.); 11 Unidad Clínica de Enfermedades Infecciosas y Microbiología, Hospital Universitario de Valme, 41013 Sevilla, Spain; juan.macias.sanchez@gmail.com (J.M.); anais.corgo@gmail.com (A.C.-G.); japineda@telefonica.net (J.A.P.); martaffuertes@gmail.com (M.F.-F.); lmreal67b@gmail.com (L.M.R.); 12 Servicio de Neurología, Hospital Universitario Central de Asturias, 33011 Oviedo, Spain; manuelmenendez@gmail.com; 13 Instituto de Investigación Sanitaria del Principado de Asturias (ISPA), 33011 Asturias, Spain; carmenmartrod@gmail.com (C.M.R.); irene.roa81@gmail.com (I.R.A.); victoria.alvarez@sespa.es (V.Á.); 14 Departamento de Medicina, Universidad de Oviedo, 33011 Oviedo, Spain; 15 Department of Neurology, University Hospital La Paz-Universidad Autónoma-Madrid, 28046 Madrid, Spain; 16 University Hospital La Paz Research Institute (IdiPaz), 28029 Madrid, Spain; 17 Fundació Docència i Recerca MútuaTerrassa, 08221 Terrassa, Spain; monicadifa@gmail.com (M.D.-F.); ignacio.alvafer@gmail.com (I.A.); pastorpau@gmail.com (P.P.); 18 Memory Disorders Unit, Department of Neurology, Hospital Universitari Mutua de Terrassa, 08221 Terrassa, Spain; 19 Neurology Service, Marqués de Valdecilla University Hospital (University of Cantabria and IDIVAL), 39008 Santander, Spain; 20 Hospital Universitario Ramon y Cajal, IRYCIS, 28034 Madrid, Spain; sebastian.garcia@salud.madrid.org (S.G.-M.); ggribas@salud.madrid.org (G.G.-R.); 21 Banco de Tejidos de la Fundación CIEN, 28034 Madrid, Spain; 22 Hospital de Cabueñes, 33394 Gijón, Spain; 23 Unidad de Trastornos del Movimiento, Servicio de Neurología y Neurofisiología, Instituto de Biomedicina de Sevilla (IBiS), Hospital Universitario Virgen del Rocío/CSIC/Universidad de Sevilla, 41013 Sevilla, Spain; 24 Laboratorio de Genética, Hospital Universitario Central de Asturias, 33011 Oviedo, Spain; 25 Alzheimer Research Center & Memory Clinic, Andalusian Institute for Neuroscience, 29012 Málaga, Spain; silviamendoza@gmail.com; 26 Department of Neurology/CIEN Foundation/Queen Sofia Foundation Alzheimer Center, 28220 Madrid, Spain; tdelser@fundacioncien.es; 27 Centro de Biología Molecular Severo Ochoa (UAM-CSIC), 28049 Madrid, Spain; 28 Instituto de Investigacion Sanitaria ‘Hospital la Paz’ (IdIPaz), 28029 Madrid, Spain; 29 Universidad Autónoma de Madrid, 28049 Madrid, Spain; 30 Department of Brain Sciences, Imperial College, London SW7 2BX, UK; 31 Department of Neurology, Pontificia Universidad Católica, Santiago 340, Chile; 32 Fundación Pública Galega de Medicina Xenómica-CIBERER-IDIS, 15705 Santiago de Compostela, Spain

**Keywords:** SARS-CoV-2, COVID-19, GWAS, GR@ACE/DEGESCO, dementia, *APOE*

## Abstract

Emerging studies have suggested several chromosomal regions as potential host genetic factors involved in the susceptibility to SARS-CoV-2 infection and disease outcome. We nested a COVID-19 genome-wide association study using the GR@ACE/DEGESCO study, searching for susceptibility factors associated with COVID-19 disease. To this end, we compared 221 COVID-19 confirmed cases with 17,035 individuals in whom the COVID-19 disease status was unknown. Then, we performed a meta-analysis with the publicly available data from the COVID-19 Host Genetics Initiative. Because the *APOE* locus has been suggested as a potential modifier of COVID-19 disease, we added sensitivity analyses stratifying by dementia status or by disease severity. We confirmed the existence of the 3p21.31 region (*LZTFL1, SLC6A20*) implicated in the susceptibility to SARS-CoV-2 infection and *TYK2* gene might be involved in COVID-19 severity. Nevertheless, no statistically significant association was observed in the COVID-19 fatal outcome or in the stratified analyses (dementia-only and non-dementia strata) for the *APOE* locus not supporting its involvement in SARS-CoV-2 pathobiology or COVID-19 prognosis.

## 1. Introduction

The coronavirus disease [1] 2019 (COVID-19) has provoked a global crisis. This respiratory infection is caused by the severe acute respiratory syndrome coronavirus 2 (SARS-CoV-2) discovered in Wuhan, China, in late 2019. Due to the rapid evolution to a pandemic, insights into how to better understand and combat COVID-19 are desperately needed.

Compared to clinical [2,3,4] or epidemiological [5] characteristics of the disease, the role of host genetic factors affecting the susceptibility and severity of the COVID-19 disease has been poorly studied to date. Given the importance and urgency of understanding such factors, the ‘COVID-19 Host Genetics Initiative [3]’ (COVID-19 HGI) was launched. This initiative brings together the human genetics scientific worldwide community to generate, share, and analyze data relating to the genetic determinants of COVID-19 susceptibility and severity outcomes. Emerging studies include those from Ellinghaus et al. [6], who conducted a genome-wide association study (GWAS) of COVID-19 cases and identified two loci that achieved genome-wide significance: the blood type *ABO* locus on chromosome (chr) 9, and a cluster of immune functions genes on chr 3 (candidate gene *SLC6A20*). Additionally, Pairo-Castineira et al. [7] reported a genome wide significant association in a gene cluster encoding antiviral restriction enzyme activators (*OAS*) and near the *TYK2, DPP9,* and *IFNAR2* genes. Torre-Fuentes et al. [8], through whole-exome sequencing, showed an association of exonic variants in the *ACE2*, *TMPRSS2*, and *FURIN* genes in relation to the presence or absence of SARS-CoV-2 infection. Roberts et al. [9] identified three novel loci; near *IVNS1ABP/SWT1*, a gene involved in influenza virus replication associated only in males, and two genes with established roles in viral replication or immunity (*SMRR1* and the immunoglobulin lambda locus). Furthermore, an independent study using the UK Biobank [10] cohort recently suggested [11] that *APOE* ε4ε4 genotype increases the risks of severe SARS-CoV-2 infection, independent of preexisting dementia, cardiovascular disease, and type-2 diabetes. A population survey of older people in Madrid (Vallecas Project) [12] also reported a significant association of the *APOE* ε4 allele with the incidence of SARS-CoV-2 infection.

To validate emerging genetic associations relevant to COVID-19 susceptibility and severity outcomes from these recent studies, further investigation in independent datasets is needed. To investigate host factors influencing SARS-CoV-2 infection and disease outcome, we took advantage of the GR@ACE/DEGESCO cohort investigating Alzheimer’s disease (AD) and related dementias. This dataset presents the largest case-control GWAS study conducted in the Spanish population to date [13,14]. To identify subjects included in our study who were infected by the coronavirus, we prospectively requested data from all physicians monitoring the subjects included in the GR@ACE/DEGESCO dataset. The main objective of this manuscript is to communicate the novel resource to the scientific community and also to provide independent replication in candidate SNPs previously reported in the literature to date. For that purpose, the data received were organized to design nested case-control studies. Candidate SNPs observed in previous studies were evaluated. Using this approach, we were able to confirm two loci previously proposed, but no evidence was obtained about the role of *APOE* isoforms in the COVID-19 incidence or fatal outcome.

## 2. Methods

### 2.1. Study Participants

To conduct this research, we used genetic data obtained from 17,256 subjects included in the GR@ACE/DEGESCO GWAS which also includes the Vallecas project cohort [12]. Clinical definitions and methods used for genotyping and quality controls applied have been published elsewhere [13,14]. From April to October 2020, information on SARS-CoV-2 infection and COVID-19 disease outcome was collected prospectively by clinicians following up the effect of the pandemic in individuals included in the GR@ACE study. Specifically, we started monitoring the Fundació ACE patients by regular teleconferences with patients and families [15]. Briefly, we designed a telematic COVID-19 survey intended to assess exposure, risk factors, symptomatology, and demographic information associated with COVID-19 susceptibility and severity. Data obtained from these self-reported surveys were followed up by our professionals. Information retrieved from patients and families was validated against the clinical history obtained from general practitioners and hospital records. Only individuals having confirmatory evidence of COVID-19 disease in their clinical records were endorsed as COVID-19 cases for this study. Fatalities associated with SARS-CoV-2 infection were also confirmed with patient relatives. Using the information retrieved, we constructed two phenotypes: the first, in which individuals who reported a positive COVID-19 test were compared to those who reported a negative test or were without information (e.g., population), was intended to assess the susceptibility to SARS-CoV-2 infection; the other, in which people who died with COVID-19 were compared to those who were infected but survived the disease, was intended to assess the disease severity. Similarly, participants from the Vallecas Project cohort were contacted by a phone call at the end of April 2020, and received a survey to explore the incidence, clinical features, and severity of COVID-19.

### 2.2. Meta-Analyses

We used the data freeze 4 (20 October 2020) from the COVID-19 Host Genetics Initiative (HGI) (https://www.covid19hg.org/, accessed date: 1 October 2021) and conducted a meta-analysis. We downloaded the phenotypes for COVID-19 vs. Population (GRCh38 leave out 23 and Me, Susceptibility analysis C2) “ALL” population from 36 studies with 30,937 cases and 1,471,815 controls and the “EUR” population from 22 studies with 14,134 cases and 1,284,876 controls. Cases were individuals with laboratory confirmation of SARS-CoV-2 infection (RNA and/or serology based), physician confirmed or self-reported COVID-19 positive (e.g., by questionnaire). Controls were everybody who was not a case, e.g., population.

### 2.3. Sample Processing, Genotyping, Quality Control, and Imputation

DNA was extracted from peripheral blood according to standard procedures using the Chemagic system (Perkin Elmer). Samples reaching DNA concentrations of >10 ng/µL and presenting high integrity were included for genotyping. Cases and controls were randomized across sample plates to avoid batch effects.

For genotyping, we used the Axiom 815K Spanish biobank array (Thermo Fisher) at the Spanish National Centre for Genotyping (CeGEN, Santiago de Compostela, Spain). Details on genotyping and quality-control procedures are provided in previous publications [13,14]. Briefly, individuals with low-quality samples, excess of heterozygosity, sex discrepancies, and familial relations between samples (PI-HAT > 0.1875) were excluded from the analysis. A principal component analysis (PCA) was performed and population outliers were removed. Variants with call rate below 95% or deviation from the Hardy–Weinberg equilibrium (*p* ≤ 1 × 10^−6^) were also removed from the analysis. To maximize genetic coverage, we performed single-nucleotide polymorphism (SNP) imputation on genome build GRCh38 using the Trans-Omics for Precision Medicine (TOPMed) imputation server [16,17,18]. Low imputation quality variants (R^2^ < 0.30) were excluded. After QC steps, we tested 14,212,906 genetic variants for association with COVID-19 disease.

### 2.4. Targeted Phenotypes and Statistical Analyses

The primary analysis focuses on SARS-CoV-2 infection susceptibility. Individuals with a positive test for infection or suspected COVID-19 were included as cases and individuals with unknown SARS-CoV-2 infection status were used as controls for the subsequent genome-wide association studies (Table 1). The inevitable presence of individuals in the control group who may exhibit the critical illness phenotype if exposed to SARS-CoV-2 is expected to bias any associations towards the null. For that reason, any estimate on SARS-CoV-2 susceptibility must be considered highly conservative. Because COVID-19 is highly dynamic in the Spanish population, we plan to update GWAS information every six months. Periodic data releases will be uploaded to the GR@ACE/DEGESCO website and will be publicly available to registered users.

The *APOE* locus is the most important genetic risk factor for AD [19,20]. Beyond its involvement in dementia causality, the locus is also involved in a number of cardiovascular phenotypes [21] and human longevity [22,23] of special interest for dementia studies like GR@ACE/DEGESCO. Because the *APOE* locus has been suggested as a potential modifier of COVID-19, we decided to add stratified analyses by using dementia-only and non-dementia strata. Meta-analysis of both strata was performed to assess heterogeneity. In addition, point estimates were obtained using only infected populations (survival analysis). For fatal COVID-19 disease outcome, the population was restricted only to those subjects with confirmed SARS-CoV-2 infection. Then, the case status was dichotomized as COVID-19 exitus and non-exitus. GWAS were performed separately for both phenotypes (SARS-CoV-2 susceptibility and fatal COVID-19 disease outcome). Logistic regression unadjusted models were fitted using Plink (v2.00a). To maximize the statistical power and because of the difference between the cases/control sample sizes, we decided to conduct the primary analyses in selected candidate SNPs without any covariate in the model. Later, we applied linear mixed models (LMM) to the significant signals to see if the effect is altered by the impact of the covariates using lme4 package in R. *APOE* locus involvement in fatal COVID-19 disease outcome was also evaluated. Because of the strong association of *APOE* with Alzheimer’s disease and longevity, its involvement was adjusted for age and dementia status. Subsequently, we performed two fixed-effects inverse-variance–weighted meta-analyses with METAL [24] on the summary statistics of the GR@ACE/DEGESCO study and the COVID-19 HGI (ALL and EUR populations) to maximize the statistical power. We selected 30 variants in 17 genome-wide candidate regions from five recent publications described in Table S1.

## 3. Results

We obtained point-effects for the entire human genome by measuring the effect of each SNP variant studied on susceptibility to SARS-CoV-2 infection and COVID-19 disease survival in the GR@ACE/DEGESCO cohort (Table 1). Genomic inflation factors (λ) of the models ranged between 0.995 and 1.017 (Table S1). These calculations suggest no gross bias or stratification issues in any comparison conducted. Because of the small number of individuals with SARS-CoV-2 infection in our study (n = 221), we were unable to identify any GWAS significant signal using GR@ACE/DEGESCO data alone. GWAS summary statistics for the entire genome are available and can be incorporated into ongoing international efforts.

On the basis of previously published GWAS results related to COVID-19 host susceptibility analyses [6,7,8,9,25], we selected thirty lead SNPs for independent replication in the GR@ACE/DEGESCO data (Table S1). Importantly, 9 of 30 SNPs were validated in at least one GWAS comparison. Interestingly, despite the small sample size, we observed a fully independent replication in chr3 locus near the *LZTFL1* (rs71325088 COVID-19 fatal outcome analysis; OR = 3.03 (1.24–7.45), *p*-value = 0.015) and *SLC6A20* genes (rs11385942 COVID-19 fatal outcome analysis; OR = 2.34 (1.03–5.35), *p*-value = 0.043). This result provides independent evidence of the role of this specific SNP in COVID-19 fatal outcome. In the case of *TYK2* gene in chr19 (rs74956615), we observed a compatible point effect, direction and statistical significance for susceptibility (OR = 1.70 (1.13–2.55), *p*-value = 0.011), COVID-19 fatal outcome (OR = 4.96 (2.02–12.20), *p*-value = 4.85 × 10^−4^), an improvement in the meta-analysis (EUR, OR = 1.11 (1.04–1.19), *p*-value = 1.63 × 10^−3^; All, OR = 1.11 (1.04–1.19), *p*-value = 9.92 × 10^−4^, Supplementary Table S2) and confirmation in the linear mixed models (*p*-value = 5.5 × 10^−4^, Supplementary Table S3).

In addition, three candidate SNPs previously reported displayed compatible effect size and direction (*rs9380142-HLA-G, rs3131294-NOTCH4,* and *rs10735079-OAS3*) in our datasets. Seven markers in the *SMRR1, IVNS1ABP/SWT1, CCHCR1, OAS1, TYK2,* and *IFNAR2* genomic regions have the same effect size direction, but remained non-significant. This result is suggesting a lack of power for detecting them in the COVID-19 fatal outcome analysis. Finally, the meta-analysis revealed slight improvements in *TYK2* and *IFNAR2* genes and slight reductions of significance in *HLA-G* and *ABO* genes with susceptibility to SARS-CoV-2 infection (Supplementary Table S2).

**Figure 1:**
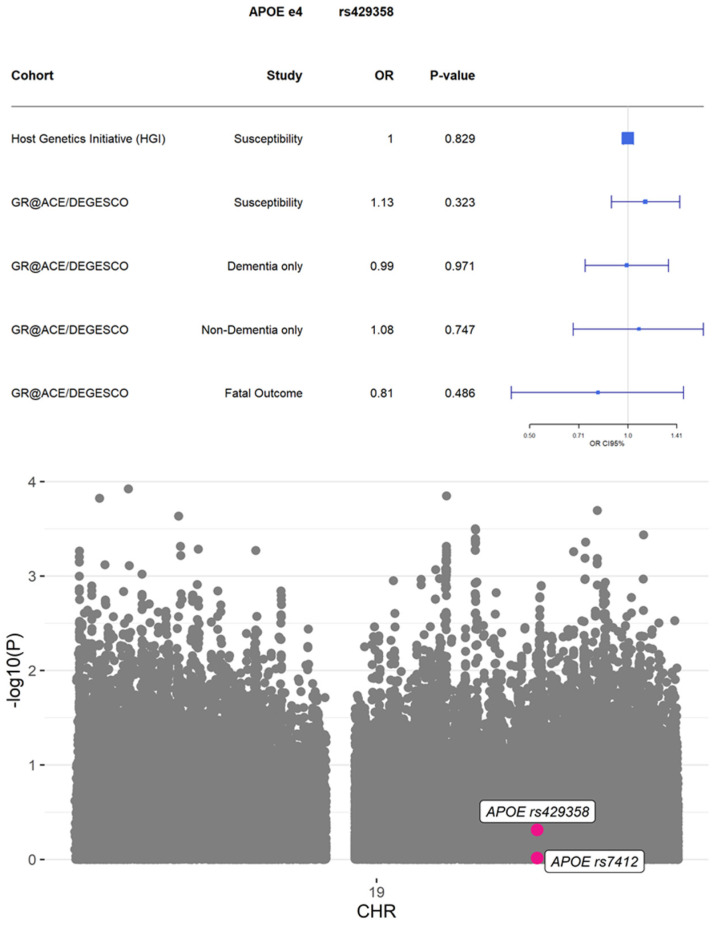
Forest plot comparing estimates for rs429358 *APOE* ε4 locus in different analysis. Manhattan plot for chr19 for fatal outcome analysis results in GR@ACE/DEGESCO population (exitus vs. non-exitus adjusted by age and dementia status; total sample size = 221). Pink dots correspond to SNPs configuring common *APOE* haplogenotypes (ε2/ε3/ε4).

In contrast, we were not be able to observe any evidence of statistically significant association, a compatible point effect, nor consistent effect direction in the COVID-19 fatal outcome analysis adjusted by age and dementia status for the *APOE* locus (rs429358, OR = 0.81 (0.44–1.48), *p*-value = 0.486; rs7412, OR = 1.02 (0.37–2.82), *p*-value = 0.967; Figure 1, Table S1), in the stratified analyses using dementia-only (rs429358, OR = 0.99 (0.74–1.33), *p*-value = 0.971; rs7412, OR = 1.07 (0.57–2.00), *p*-value = 0.838) or in non-dementia strata (rs429358, OR = 1.08 (0.68–1.70), *p*-value = 0.747; rs7412, OR = 1.29 (0.75–2.22), *p*-value = 0.365). Further meta-analysis of dementia and non-dementia groups indicated an absence of heterogeneity among strata. Furthermore, HGI consortium [25] data alone and a susceptibility meta-analysis with both HGI and GR@ACE/DEGESCO confirmed our findings (meta-analysis EUR, rs429358, OR = 1.03 (0.99–1.07), *p*-value = 0.199; rs7412, OR = 0.99 (0.94–1.04), *p*-value = 0.574, Supplementary Table S2).

## 4. Discussion

Our data strengthen the evidence for an association with SARS-CoV-2 susceptibility at the chr3p21.31 gene cluster, first identified by Ellinghaus et al. [6] and recently validated by others [26,27]. In particular, *SLC6A20* can be linked to the association via eQTLs within breast epithelium and esophagus muscularis mucosa according to GTEx [28], suggesting increased expression of *SLC6A20* correlates with increased risk of severe outcomes. Further, *SLC6A20* can form a complex with angiotensin converting enzyme 2 (ACE2), the cell surface receptor for SARS-CoV-2 viral entry [29]. Thus, it is possible that increased *SLC6A20* expression leads to increased ACE2 protein levels and greater viral uptake. Other candidates from the region such as *LZFTL1 (CCR9, CXCR6, XCR1, FYCO1)* have been implicated in respiratory functions and/or in the immune system that should be considered and investigated.

A potential causal role for *TYK2* was also statistically significant in our analysis. These findings support the results reported by Pairo-Castineira et al. [7] in which critical illness in COVID-19 is related to host-driven inflammatory lung injury, which is a key mechanism of late and life-threatening COVID-19 disease.

The GR@ACE/DEGESCO dementia configuration allows us to study, in a different population, the *APOE* effect suggested by previous studies with the UK Biobank [10] on COVID-19 disease or in a Spanish elderly cohort [12]. Kuo [10] et al. reported that the *APOE* ε4ε4 genotype was associated with 2.2-fold increased risks of test positivity and of 4.3-fold more mortality after testing positive relative to *APOE* ε3ε3 individuals, suggesting that the *APOE* ε4ε4 genotype represents a significant risk for the development of severe COVID-19, as well as death following infection. Del Ser [12] et al. reported a significantly higher incidence of COVID-19 disease in the individuals carrying an *APOE* ε4 genotype. The effect of age [30] on the severity of COVID-19 disease and *APOE* [31] in dementia is well known. Therefore, controlling these factors is essential to have more reliable and accurate results. Interestingly, our stratification in dementia, non-dementia, and fatal COVID-19 disease outcome analysis adjusting by age and dementia diagnosis does not corroborate the previous results, suggesting a lack of association of *APOE* with COVID-19 outcome. Despite our findings, the effect of *APOE* in SARS-CoV-2 infection and its lethality deserves further investigation. Of 40 genes previously identified as crucial for SARS-CoV-2 infection, two, the endosomal entry receptor ACTR2 and the ATP6AP2 ATPase, are involved in endosome function [32]. Intriguingly, this molecular pathway has been prioritized as an important causal pathway associated with AD [33]. Curiously, *ACTR2* and *ATP6AP2* genes are more highly expressed in ApoE4 astrocytes, which have larger early endosomes than do ApoE3 cells [32]. More research is needed to determine the real impact of *APOE* alleles in COVID-19 morbid-mortality and host resistance to SARS-CoV-2 infection.

The investigation of host factors affecting SARS-CoV-2 infection susceptibility and lethality may benefit from dementia-only GWAS studies. For example, exploring genetics elements in populations with more homogenous comorbid profiles might offer enhanced precision for studying *APOE* involvement in COVID-19 risk and prognosis. Hence, we propose to nest COVID-19 studies in large-meta-GWAS for AD such as GR@ACE/DEGESCO or the European Alzheimer Disease Biobank (EADB; Bellenguez et al. [34]). Dementia-only studies could be very important in disentangling potential *APOE* effects that might be obscured by selection bias affecting SARS-CoV-2 exposure and infection outcome. We feel that dementia-only studies would be more resistant to obvious selection bias potentially affecting people with dementia during the COVID-19 crisis. On one hand, people with dementia may have an increased risk of infections because of their lack of autonomy for configuring self-protection measures. They have also an increased risk of being in nursing homes [35]. On the other hand, in practice, people with dementia may have had less chance of surviving because of potential discrimination in intensive care units during pandemic peaks [36,37]. Furthermore, comorbidities typically associated with dementia might also contribute to the increased morbidity–mortality observed in SARS-CoV-2 infected individuals belonging to this vulnerable population [38].

The main limitation of the study is the small number of patients in the cohort with SARS-CoV-2 infection, which reduces the statistical power of the results and limits the possibility of evaluating other aspects of COVID-19 that could be influenced by genetics, including the severity of the infection. Another limitation is the lack of confirmation of SARS-CoV-2 infection in many cases: for a considerable period during the pandemic, the Spanish healthcare authorities recommended that infected patients be quarantined without PCR confirmation of the infection. This limitation might have contaminated the control groups of our study further reducing the power to detect genuine signals associated to susceptibility to SARS-CoV-2 infection and COVID-19 development, but not to disease mortality analyses conducted. Despite these considerations, we have been able to validate some signals and the strengths of the study provide relevant information to *APOE* and COVID-19 disease. For future studies, it would also be interesting to be able to compare the effects caused between the SARS-CoV-2 variants.

In conclusion, we confirmed the 3p21.31 region (*LZTFL1*, *SLC6A20*), *TYK2* gene as a genetic susceptibility locus involved in SARS-CoV-2 infection using an independent Spanish dataset. With COVID-19 HGI meta-analysis, other suggested regions were reinforced. In contrast, our Dementia-only GWAS study did not support an *APOE* locus involvement in SARS-CoV-2 pathobiology or COVID-19 prognosis. To increase the statistical power of this study, we continue monitoring COVID-19 disease in the GR@ACE/DEGESCO participants.

## Figures and Tables

**Table 1:** Demographics of the GWAS models in GR@ACE/DEGESCO cohort.

Whole Study			
	Cases with COVID-19	Population	Total
Participants (*n*)	221	17,035	17,256
Gender (% Female)	69.68	58.78	58.92
Age, years (SD)	71.08 (17.55)	70.45 (16.49)	70.46 (16.51)
*APOE4* carriers (%)	34.39	30.13	30.19
Dementia (%)	57.92	45.79	45.95
Dementia individuals			
	Cases with COVID-19	Population	Total
Participants (*n*)	128	7801	7929
Gender (% Female)	78.12	68.43	68.58
Age, years (SD)	75.4 (17.29)	72.86 (14.94)	72.93 (15.01)
*APOE4* carriers (%)	42.19	41.35	41.37
Non-Dementia individuals			
	Cases with COVID-19	Population	Total
Participants (*n*)	93	9223	9316
Gender (% Female)	58.06	50.61	50.69
Age, years (SD)	65.16 (16.21)	69.05 (17.17)	69 (17.16)
*APOE4* carriers (%)	23.66	20.64	20.67
Individuals with COVID-19			
	Cases with COVID-19	Population	Total
Participants (*n*)	55	166	221
Gender (% Female)	61.82	72.29	69.68
Age, years (SD)	83.44 (7.83)	76.68 (15.35)	78.42 (14.11)
*APOE4* carriers (%)	36.36	33.73	34.39
Dementia (%)	87.27	50.60	59.73

## Data Availability

The data that support the findings of this study are available from the corresponding author upon reasonable request.

## Supplementary Materials

The following are available online at https://www.mdpi.com/article/10.3390/jpm11121318/s1, Supplementary Table S1: Table of emerging signals evaluated in GR@ACE/DEGESCO cohort. Table S2: Meta-analysis table of emerging signals for GR@ACE/DEGESCO and HGI Consortia. Supplementary Table S3: Results for linear mixed modeling (LMM) for the signals replicated in GR@ACE/DEGESCO cohort in the different analysis.
